# Adjusting advance care planning to older people’s needs: results from focus groups and interviews

**DOI:** 10.1186/s12913-023-10491-x

**Published:** 2024-01-10

**Authors:** Heike Gerger, Anneke van Dijk-de Vries, Albine Moser, Inge Jochem, Marja Veenstra, Marieke Perry, Marloes van Bokhoven

**Affiliations:** 1 Department of Family Medicine, Care and Public Health Research Institute, Maastricht University, Maastricht, the Netherlands; 2 Research Centre Autonomy and Participation of Chronically Ill People, Zuyd University of Applied Sciences, Heerlen, The Netherlands; 3 MCC Omnes, Sittard, the Netherlands; 4 Burgerkracht Limburg, Sittard, the Netherlands; 5 Department of Primary Care Medicine and Geriatrics, Radboudumc, Nijmegen, the Netherlands

**Keywords:** Mixed-methods, Participatory research, Advance care planning, Personalized care, ACP

## Abstract

**Background:**

Advance care planning (ACP) is becoming increasingly important in medical care. Some suggest standardized approaches to initiate ACP with all older adults. However, the idea of patient-centered care suggests more nuanced approaches tailored to individual older adults’ needs. This study investigated how older adults with different views and needs about ACP can be approached in an adequate and most beneficial way by health care professionals.

**Methods:**

We used questionnaires, interviews, focus groups and informal conversations with older adults, living in their own homes, who volunteered to take part in our research. The research was participatory as we collaborated closely with practice partners and we used the obtained findings immediately and continuously to inform the next steps of our research throughout the process.

**Results:**

We identified three subgroups of older adults with differential needs regarding ACP-related activities: The first group avoids talking about their needs and wishes for care towards the end of life. These older people benefit from activities, which aim at motivating them to concern themselves with ACP-related topics. The second group consists of older adults who are in principle open for ACP-conversations but do not initiate these themselves. This group either trusts their next-of-kin or their healthcare professional to act in accordance with their wishes or does not bring up the topic in order to avoid confronting relevant others with possibly unpleasant topics. This group of people benefits from information about ACP and from healthcare professionals initiating the ACP process. The third group of older people initiates the ACP process themselves, gathers information, and takes the necessary steps for ACP. With this group it remains relevant to check carefully whether they have indeed taken all relevant steps and shared the information with all relevant involved care institutions and relatives.

**Conclusions:**

We propose a model to simplify adjustments of ACP to individuals’ needs. Our suggested approach might contribute to increasing the motivation of older people to engage in ACP conversations if these are more closely related to their own needs. Further, it might also contribute to simplifying the individual shaping of the ACP process for healthcare professionals as our suggested model offers clear guidance for approaching different types of older people in different ways. The suggested approach may in future be used for training health care professionals in the conduct of ACP conversations.

**Supplementary Information:**

The online version contains supplementary material available at 10.1186/s12913-023-10491-x.

## Introduction

In a world of aging societies, advance care planning (ACP) has become an important point on health care agendas [1]. ACP describes a process which aims at improving quality of life towards the end of life and which may involve individuals, their health care professionals, and their next-of-kin [2–4]. For instance, a panel of 109 international experts suggest the following definition: “ACP is considered to be a process that includes the identification of values and defining goals and preferences for future medical treatment and care and discussion of these factors with the patient’s family and health-care providers [2].” Potential benefits of ACP include increased compliance with older peoples’ end-of-life wishes, increased satisfaction of older people and their families, as well as reduction of stress, anxiety and depression [5–8].

Conversations about ACP are by nature very personal. They require decisions which many people consider difficult, emotionally demanding, or irrelevant for their life [9]. In the Netherlands, a number of guidelines for GPs are available which describe ACP procedures [10–13]. In many cases health care professionals are advised to engage in ACP conversations with all people entering the last phase of life, e.g. above a certain age [14, 15].

However, previously, different trajectories of the last phase of life depending on the presence or absence of certain illnesses have been proposed [16, 17], suggesting that people with cancer, multimorbidity, or cognitive decline might need different ACP-related activities. In addition, depending on the personal background, including culture, personality, and previous experiences, people might need to be approached in different ways when it comes to ACP [4, 18–20]. The summarized observations are well in line with patient-centered care models, which ask for adapting procedures and treatments to individual patients’ needs [21], which in turn raises the question whether the general advice for engaging all older adults in ACP might need some refinement.

In our research, we wanted to explore whether a typology of older people can be developed which simplifies the individual shaping of the ACP process for healthcare professionals. Accordingly, our research addressed the following two research questions: (1) Can different types of older people be identified according to their views and needs about the last phase of life and ACP? (2) How can the different types of older people be approached in an adequate way by health care professionals in ACP conversations?

## Methods

### Research context and research team characteristics

We conducted our research in the South of the Netherlands. This region is the unhealthiest region of the Netherlands with a relatively high proportion of people with low or average socioeconomic status and low social participation [22].

Our participatory research project was conducted in close collaboration between researchers with various backgrounds (including health sciences, psychology, general practice medicine, and nurse sciences) and regional practice partners involved in older adult care planning (i.e., “Omnes”, a regional center for organizing interprofessional medical collaboration) and in public involvement (i.e., “Burgerkracht Limburg”, a provincial network organization to facilitate citizen participation) as well as with older adults themselves. By applying a pragmatic participatory research approach we aimed to come to practical conclusions, which are close to the actual needs of our study population and carry implications for implementation in health care practice.

### Design

We used a mixed-methods approach and conducted two qualitative studies using focus groups and individual interviews. In the first study we used a questionnaire for the purposeful selection of focus group participants. Because of difficulties with recruiting participants for the planned focus groups we adapted our data collection and partly conducted individual interviews instead. In the second study individual interviews were conducted with study participants.

We triangulated data in the first study intentionally by combining the information from the questionnaires with the information from subsequent focus groups or interviews. Overall data triangulation occurred also across the two studies, as we integrated all information from study one and two in order to answer our research questions.

### Recruitment and selection of participants

**Fig. 1:**
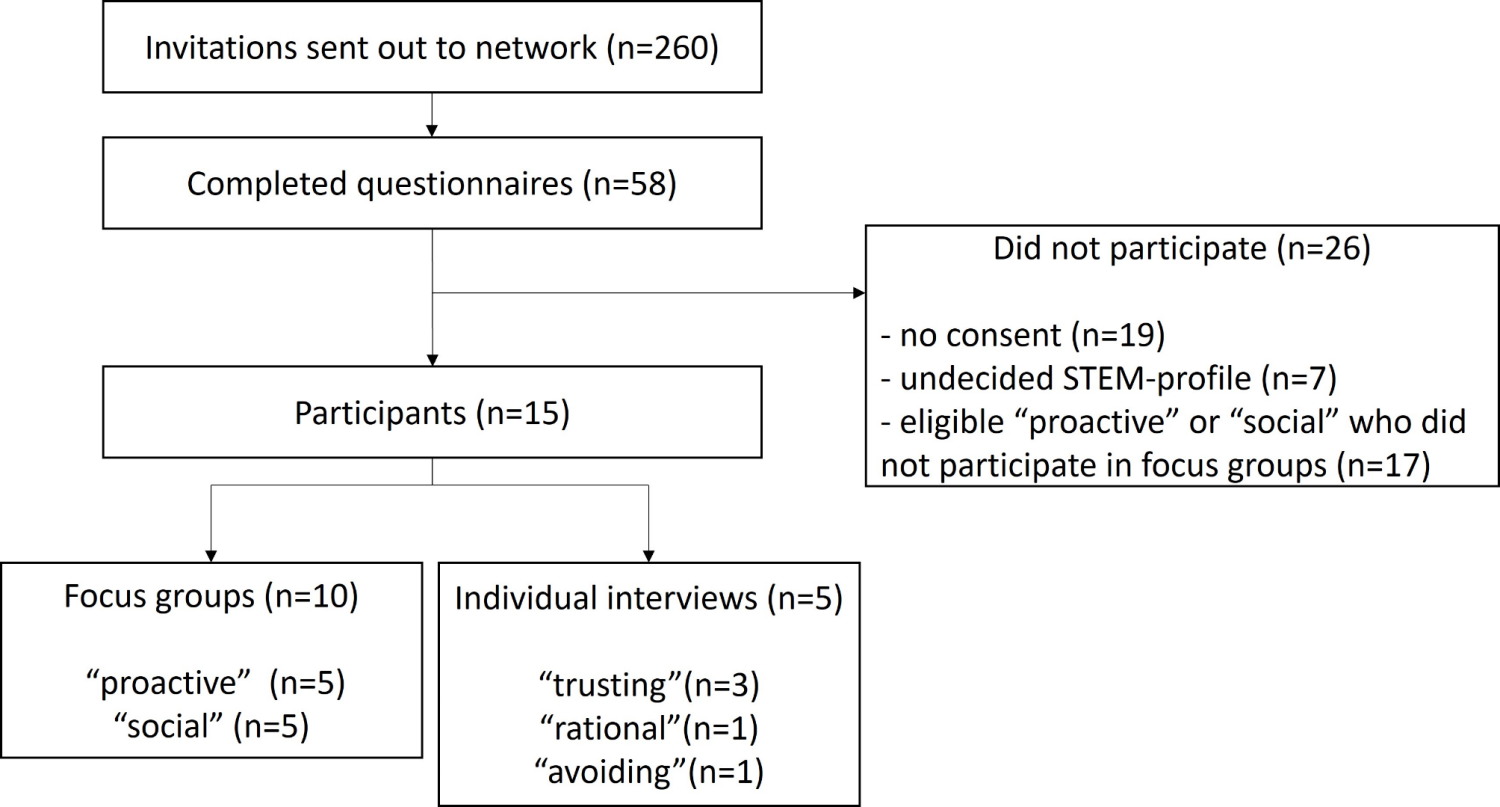
Flow chart of participant recruitment in study one

For the investigation of our first research question we recruited older people aged 70 or above, with diverse health care needs as well as social and cultural backgrounds who lived at home. Our practice partners invited older adults to participate in focus groups by using their internal networks and mailing-lists as well as by visiting guided group activities for older people (e.g., a coffee or a bingo afternoon). The process of participant recruitment of study one is presented in the flow chart in Fig. 1.

In line with the idea of purposeful sampling we used a questionnaire to identify older people with different views and attitudes towards death, the so-called STEM questionnaire, which was developed and validated based on a sample of over 1570 adult Dutch participants from the general population [23–25]. The questionnaire consists of 40 items relating to personal preferences concerning dying and the death: “dying in your own way” (in Dutch: “STerven op je Eigen Manier”). For each item, participants choose one out of four responses (i.e., disagree, mainly disagree, mainly agree, and agree). Participants’ responses to the STEM questionnaire were analyzed automatically using the algorithms elaborated by the developers of the questionnaire. The analysis of the questionnaire results concludes with providing a profile for each participant reflecting five different groups of people: 1st the proactive type (18% of the Dutch general population), 2nd the social type (33%), 3rd the trusting type (12%), 4th the rationale type (15%), 5th the avoiding type (22%). The type with the highest agreement is defined as the predominant type.

In order to investigate our second research question we approached older adults on a public regional health fair organized for informing the public about recent health trends and innovations. As one of the exhibitors on the fair we had a stand with various information material on the topic of ACP and a banner with quotes from the first study which were meant to attract the attention of older people. Two researchers approached older people passing by to engage in ACP-related conversations without applying any further inclusion criteria.

### Data collection

#### Focus groups and interviews:

In order to study the first research question, we organized guided focus group sessions in collaboration with our practice partners with 5 older adults reflecting the same STEM type as predominant type. During these sessions we discussed with the participants their ACP-related views, wishes and choices. Instead of conducting focus group sessions we collected data in individual interviews if not enough older adults reflecting the same STEM profile volunteered to be part of a focus group.

For this data collection, we developed a pre-specified session guide with questions about (A) the current living situation of older adults, (B) the role of their social network in ACP, (C) the role of their primary care physician, (D) the most important sources of information when it comes to ACP, and D) important aspects concerning care wishes (see supplementary material for the interview questions used in the focus group sessions and individual interviews).

The focus groups were led by two moderators. The collected information was noted down by one the session moderators and the sessions were audio-recorded. The interviews were conducted by one interviewer who used the interview-guide in order to note down the interviewees responses.

The collected data was used for creating descriptions of five types of older adults including prototypical ACP-related views and needs.

#### Health fair interviews:

In order to study the second research question, two researchers independently engaged in individual conversations with health fair visitors who agreed to participate in a short ACP-related interview. Using a prepared semi-structured interview form the following questions could be explored and discussed: 1^st^ with which one of the five types a person identified most, 2^nd^ which steps a person had already taken with regards to ACP, 3^rd^ what their needs were regarding the next steps in the ACP process, and 4^th^ which information sources and tools for facilitating the ACP process they would need in order to take the next step.

For questions two, three, and four we prespecified three steps in the ACP process: (A) *Motivation*: communication about the relevance of ACP and motivation to get involved with the topic; (B) *Information*: information gathering about possibilities and requirements with regards to ACP; (C) *Writing down*: writing down the own ACP-related decisions. Participants were asked to respond to questions two to four relating to these three steps. For each of the three steps we presented different leaflet material which we used in our conversations in order to explore needs for the next steps in the ACP process. This material included books about the last phase in life and ACP, information material, booklets and forms to write down ACP-related wishes and decisions. It is important to note that not all pre-specified aspects could be discussed with each of the participants. During and after each conversation the two researchers made field notes. Participants were offered a five Euro coupon for their participation.

### Analyses

In study one the focus group sessions were audiotaped and analyzed in two steps: First, the moderator of the group discussion formulated a prototypical description, based on field notes, encompassing the most relevant characteristics and views regarding ACP mentioned in the focus group sessions. In a second step, another researcher listened to the audiotapes of the focus group sessions and checked the adequacy of the prototypical descriptions, adding missing information and applying corrections if needed. The individual interviews were not audiotaped because the interviewees did not give consent for audiotaping the sessions. Here, the interviewer formulated the description of the prototypical needs of the interviewed older adults based on field notes.

In study two, the data gathered on the health fair were transferred to an excel spreadsheet and were analyzed quantitatively and qualitatively. Here, our goal was to check whether we could identify a need for differential communication strategies about ACP in the five types of older people, which we had identified in study one. For each of the five types of older adults we calculated frequencies of the three pre-specified ACP-related activities (i.e., motivation, information, and writing down). In addition, we analyzed our free comments on the prepared forms to see whether people mentioned additional needs for ACP conversations.

Based on the typology of older people which we developed in study one, we triangulated quantitative and qualitative results from study two in order to formulate possible communication strategies which could contribute to simplifying the individual shaping of the ACP process for healthcare professionals.

## Results

In total we analyzed data from 70 conversations with older adults. Table 1 shows characteristics of the study participants and gives an overview over the source of data per study.

**Table 1:** Description of study participants

Datasource	*n*	Sex	Age
**Study one:**	**Participants:**		
Focusgroups (FG)^a^	Total: 10	Total: 6 M; 4 F	mean: 73,5
FG1 (proactive)	5	3 M; 2 F	mean: 71
FG2 (social)	5	3 M; 2 F	mean: 76
Interviews (II)^a^	Total: 5	Total: 3 M; 2 F	mean: 74
II1 (trusting)	1	F	76
II2 (trusting)	1	M	69
II3 (trusting)	1	M	82
II4 (rational)	1	F	70
II5 (avoiding	1	M	73
**Study two**:	**Conversations**:		
Health fair (HFI)^b^	Total: 55	Total: 16 M; 32 F; 6 x M + F; 1 x F + F	NA

*Note.*^a^ participants were selected based on views regarding death and dying (STEM profile), ^b^ participants invited among health fair visitors (self-selection), F = female, FG = focus group, HFI = health fair interview, II = individual interview, M = male, NA = not assessed

### Subdividing older adults into groups with differential ACP-related views and needs

Via the practice partners we included 10 older adults for the planned focus groups investigating research question one. As invitations were spread using a snowballing approach we do not have exact data regarding the total number of people contacted, neither regarding reasons for rejecting participation.

We conducted one focus group with five older adults reflecting the *proactive* profile and one focus group with five older adults predominantly reflecting the *social* profile, according to the STEM profiles. For the other three profiles (i.e., the *rational*, the *trusting*, and the *avoiding*) we conducted individual interviews with one person at the time.

An important finding was that most people did not clearly reflect one of the STEM-profiles, but rather reflected a mix of two to five different profiles. Our analyses of field notes and focus group and interview transcripts resulted in prototypical descriptions of five different types of older adults. We used the five STEM profiles as a basis and added the additional information gathered in the focus groups and interviews focusing specifically on ACP related views and needs.

On the health fair we talked with 55 older adults about ACP-related topics. Of those participants, 33 older people took the time to read the five prototypical descriptions of older adults with differential ACP-related views and needs which were developed following the focus groups and interviews in study one. With these participants we engaged into longer conversations specifically exploring ACP-related views, experiences and needs referring to one of those five types which the person most identified with. 22 of the 33 participants identified themselves with the *proactive* type (67%), four with the *social* type (12%), four with the *avoiding* type (12%), three with the *trusting* type (9%), and none self-identified as *rational type* (0%). Across these five types we identified three different needs regarding ACP-related activities (see next section and Table 2).

Of the 55 older adults 38 informed us about their current status in the process of ACP: 32% indicated they had done “everything” already, 45% indicated that they needed to record their preferences (“writing down”), 18% indicated that they needed more information (“information”) and 5% indicated that they would need to be motivated externally to initiate ACP (“motivation”), for instance by attending activities to raise the awareness for ACP. It is important to keep in mind that the majority of people we talked with (67%) self-identified as *proactive* regarding ACP.

### Identifying adequate ACP-related communication strategies per group

When integrating our results from the focus groups, interviews, and from the health fair, we identified three typical types of reactions regarding ACP conversations across the five initially identified types of people: First, a number of people avoid ACP as a topic. These people often identify themselves with descriptions of the *rational*, *avoiding* or *trusting* types of older people. A second group of people showed no or very little motivation for initiating ACP-related activities, while they did not actively avoid ACP-related topics. People in this group often identified with the descriptions of the *social* and *trusting* types of older people. Finally, a third group of people, those identifying with the *proactive* type of older people, were very open in ACP-related conversations and reported having initiated the ACP process already, or even “having done everything” already (see Table 2 for an overview of tailored communication strategies).

The *avoidant* group of people avoids talking and/or thinking about ACP. This is because either it does not seem relevant for this group in their current life or some people of this group perceive it as a taboo (see Table 3 for quotes reflecting the views of the different types of older people). The *avoidant* group was most difficult to motivate for participation in our research. The few people reflecting this group who we spoke with did mostly not show interest in communication about the topic. However, one person who self-identified with the *avoiding* type mentioned that he/she might be interested to watch some short film or to visit an informal meeting about the topic, reflecting the first of the three steps in the ACP process (i.e., motivation).

The second group of people is *not motivated* to initiate ACP. However, these people do not find it a taboo, neither do they actually avoid talking about this topic. In this group, ACP conversations do not happen, because the older adults think that either their care network or friends and family do know what they wish for the future, or they do not want to bother others with their issues, or because no one seems to take the initiative for such a conversation. With regards to the three steps within the ACP process, this group of people was interested in receiving help with writing down their wishes and ACP-related decisions, reflecting the third step in the ACP process (i.e., writing down). They also often lacked detailed information about possible ACP-related decisions, reflecting the second step (i.e., information).

The third group of people are *proactive* concerning ACP. In this group of people many initiate talking about the last phase of life or have taken some sort of action already to write down their last will, their wishes for future care, or restrictions to possible future treatments (e.g., deciding against cardiopulmonary resuscitation (CPR)). Importantly, when asking in-depth questions knowledge gaps became apparent sometimes, for instance concerning the relevance of sharing ACP-related decisions with the general practitioner and all relevant health care institutions (e.g., in addition to having their last will stored with a lawyer). This group of people was interested in activities related to all three steps of the ACP process (i.e., motivational activities, information, and writing down wishes and decisions).

**Table 2:** Description of three types of older adults with different views and needs regarding ACP.

Type of older adult (STEM profile)	1^st^ group: avoidant (rational, avoiding, partly trusting)	2^nd^ group: not motivated (social, partly trusting)	3^rd^ group: proactive (proactive)
**Reactions of different types of older people with regards to ACP:**	Avoid talking about ACP & last phase of life.	Do not initiate ACP and talking about last phase of life. Do not see necessity of ACP.	Initiate ACP process. May lack relevant information.
**Most relevant ACP strategy for health professionals:**	Motivation	Information	Writing down
**Implications for practice: next steps in ACP process:**	• Sensitizing for relevance of ACP. • Motivating to take initial steps in ACP process. • Involving relatives or other care givers.	• Offering information about ACP. • Motivating to take next steps in ACP process. • Recording ACP-related decisions. • Sharing ACP decisions with relevant health care institutions and relatives.	• Checking whether ACP is complete and up-to-date. • Sharing ACP decisions with relevant health care institutions and relatives.

*Note*. ACP = advance care planning

**Table 3:** Quotes reflecting differential views of three types of older people with different reaction towards ACP.

Type of older adult	Quote (Source)*
**1**^**st**^ **group**: **avoiding ACP**	“*Life is still far too much fun to think about death right now.”* (II5) *“I think about it often, because it will happen any day now…. After two seconds I quickly think of something else.”* (II4)
**2**^**nd**^ **group**: **not motivated for ACP**	*“Care decisions may be made by my loved ones when I am no longer able to do so myself.”* (II1) *“I did it [ACP] because my children asked for it. I would not have done the first steps myself, however now I find it very relaxing to know what to expect.”* (HFI: person identifying with social profile)
**3**^**rd**^ **group**: **proactive with initiating ACP**	*“I enjoy exchanging thoughts and feelings on this subject.”* (FG1) *“I want to be able to trust that a healthcare professional knows my wishes and respects them. This is why I think about my later stage of life in time and arrange what is needed.”* (FG1)

*Note*. ACP = advance care planning; FG1 = focus group proactive; HFI = health fair interview; II1 = individual interview trusting; II4 = individual interview rational; II5 = Individual interview avoiding; *Quotes were translated from Dutch to English using DeepL (www.deepl.com). The original quotes in Dutch language are available from the corresponding author upon request

## Discussion

The overarching aim of the study was to inform health professionals about possible strategies for tailoring ACP-related communication and activities to the differential needs of older adults. We expected the STEM-profiles to be a good starting point for identifying a wide range of older people with differential ACP-related views and needs. We identified three typical reactions of older adults, which require differential strategies by health professionals when communicating about ACP: The first group of older adults was not motivated to talk about the last phase in life, a second group was in principle willing to talk about the last phase of life but would not initiate ACP, while a third group willingly initiated and engaged in ACP-related activities.

### Previous research and future directions

Previous research described ACP as a process, which needs to be adapted to each individual patient [3, 26, 27]. Our findings refine general calls for health professionals to initiate an ACP conversation in a systematic and standardized way, for instance, with all older adults of a certain age [14]. Our results complement previous findings [4, 16–20] in highlighting the relevance of considering an older person’s personal views regarding ACP and of the ACP-related actions which the person has already taken. This way, not all ACP-related activities in the process (i.e., motivation, information, and writing down) need to be done with every older person. For instance, a *proactive* person might not need additional information about relevant ACP-related decisions but might only need support with sharing their own decisions with relevant health care institutions. We assume the suggested tailored approach to be more time-efficient than a standardized ACP approach, which will possibly increase the willingness of health professionals in initiating ACP conversations, and in turn the number of ACP conversations. We suggest that testing these assumptions should be subject of future research.

### Implications for practice

Our research identified three different types of people with differential ACP-related needs within the population of older adults.

With the first group of older adults who avoid ACP the necessity and potential benefits of ACP need to be discussed with the goal to at least initiate the ACP process. Importantly, this group should not be neglected as it could be reflecting the majority of people: according to a study conducted with 205 older general medicine patients from San Francisco 84% of participants reported to find ACP irrelevant [9]. With this group of older people, it might be helpful to involve relatives or other caregivers to help to motivate an older adult to get concerned with ACP. Most likely, they will not yet be ready to make concrete decisions about ACP. The first step, however, will be to initiate the ACP process.

The second group of older adults who are not motivated to initiate ACP could be approached by their care network providing relevant information if needed and suggesting to start writing down relevant wishes and decisions with regards to the last phase of life. This group of older people might require help, however. In the study conducted in San Francisco, for instance, 36% of participants endorsed information needs and 29% endorsed problems with advance directives [9]. These people might also need information regarding the relevance of sharing ACP-related decisions with their general practitioner and with all relevant health care institutions.

Finally, in ACP-related conversations with the third group, who are *proactive* regarding ACP, it might seem that no activity is needed by health professionals, as the proactive older people often respond that they have taken care of “everything” already. It remains nevertheless relevant to check whether all relevant information has indeed been written down and shared with all involved health care professionals and with the relevant relatives. Further, it needs to be checked on a regular basis whether the previously recorded decisions are still up to date.

### Strengths & limitations

Our research has some strengths. Our research team consisted of members with diverse backgrounds and working in research, in teaching, and in the practical field. This combination motivated a vivid exchange of perspectives and contributed to results with high implications for clinical practice. We see this as a strength of our research. Further, we made a big effort to identify older adults with broad and differential views on ACP-related activities and conversations. Even if the distribution of people across the different STEM profiles was not as expected, we consider it a strength of our study that we managed to include people with all of the five STEM profiles in our research.

Our study has also limitations. It is important to note that we did not use a representative sample of Dutch older adults in our research. Because of the frailty of some older people and the potential heaviness of the topic, our partners in public involvement advised against a broad recruitment strategy, for instance inviting all older people in a certain region via diverse channels, like the GP practices, pharmacies, the media or by actively contacting older people during their daily activities (e.g. shopping). As a result, despite applying diverse routes for participant recruitment, we were able to recruit only very few *avoidant* participants and the biggest response for participation in our research came from *proactive* older people. This observation carries important implications for research with older people over ACP: If research with older people is done with convenience samples, without taking into consideration the type of person, results might be biased, because of the overly large proportion of *proactive* people volunteering to participate in this type of research. Thus, our conclusions regarding the differential needs of the *avoidant* type of people concerning ACP-related activities need to be interpreted with caution and require confirmation in future studies.

We did not test for the discriminatory value of the STEM profiles. In fact most people in our sample reflected more than one STEM profile. Further, also with regards to the descriptions which we developed in study one, quite a proportion of participants in study two found it difficult to clearly identify with one prototypical description. For the purpose of our research as well as for implementing our suggestions in clinical practice, the discriminatory value of the typology is not of great relevance. We rather see the typology of three different groups of older adults with differential ACP-related needs as indicator for a possible starting point for health care professionals when initiating ACP conversations with older adults. The identification of a certain older person as belonging to one or the other type of older people should, thus, rather be considered as a starting point guiding the future ACP process, then as a goal in itself.

We used a participatory pragmatic approach, which did not reflect a highly standardized research approach. Due to the nature of the topic and the fact that we were interested in exploring the views of older adults with high relevance for practice, we decided that this approach would be the best-suited approach for our research. This decision was done in close cooperation with the involved institutions of older adults care planning and public involvement who were partners in our project from the very beginning of project planning and throughout the entire project period.

## Conclusions

ACP is relevant but initiating this process oftentimes seems difficult, either for health care professionals, the older adults, or both. In order to facilitate ACP processes, we suggest that health care professionals use the suggested typology of three different groups of older people with differential ACP related needs when initiating ACP-related conversations, which can easily be done by asking older adults about their actual needs, views, and previous experiences with ACP. Health professionals should then tailor their communication strategy to the actual needs and views of an older adult, focusing on either motivation with the *avoidant* type of older people, or on information, writing down, and sharing with the *not motivated* type, or on checking and sharing with older people of the *proactive* type. By following the suggested person-centered ACP approach, health care professionals can easily choose communication strategies which fit the needs of different types of older people.

### Electronic supplementary material

Below is the link to the electronic supplementary material.

Supplementary Material 1

Supplementary Material 2

## Data Availability

All study materials (i.e., material for recruitment, the guides for the focus groups and interviews, descriptions of the prototypical needs of older adults with regards to ACP, as well as the interview guide for the health fair) were developed for the purpose of this research in Dutch language. This study material is available from the corresponding author, upon reasonable request. In agreement with the informed consent we obtained from participants, no data will be shared.

## References

[1] World Health Organization (2018). Fact sheet: Ageing and Health.

[2] Rietjens JA, Sudore RL, Connolly M, van Delden JJ, Drickamer MA, Droger M (2017). Definition and recommendations for advance care planning: an international consensus supported by the European Association for Palliative Care. Lancet Oncol.

[3] Institute of Medicine. Dying in America. Improving Quality and Honoring Individual Preferences Near the End of Life-The Pressing Need to Improve End-of-Life Care. 2014.

[4] Gjerberg E, Lillemoen L, Førde R, Pedersen R (2015). End-of-life care communications and shared decision-making in Norwegian nursing homes - experiences and perspectives of patients and relatives. BMC Geriatr.

[5] Wright AA, Zhang B, Ray A, Mack JW, Trice E, Balboni T (2008). Associations between end-of-life discussions, patient mental health, medical care near death, and caregiver bereavement adjustment. JAMA.

[6] Houben CH, Spruit MA, Groenen MT, Wouters EF, Janssen DJ (2014). Efficacy of advance care planning: a systematic review and meta-analysis. J Am Med Dir Assoc.

[7] Brinkman-Stoppelenburg A, Rietjens JA, van der Heide A (2014). The effects of advance care planning on end-of-life care: a systematic review. Palliat Med.

[8] Detering KM, Hancock AD, Reade MC, Silvester W (2010). The impact of advance care planning on end of life care in elderly patients: randomised controlled trial. BMJ.

[9] Schickedanz AD, Schillinger D, Landefeld CS, Knight SJ, Williams BA, Sudore RL (2009). A clinical Framework for improving the Advance Care planning process: start with patients’ self-identified barriers. J Am Geriatr Soc.

[10] Landelijke Adviesgroep Eerstelijns Geneeskunde voor Ouderen. Toolkit: Advanced Care Planning mbt het levenseinde. https://www.nhg.org/sites/default/files/content/nhg_org/uploads/toolkit_acp_mbt_het_levenseindeokt_2017.pdf2017.

[11] Netwerk Palliatieve Zorg Zuidoost-Brabant. Proactieve zorgplanning - praat over wensen. https://palliaweb.nl/netwerk-zuidoostbrabant/zorgverleners/proactieve-zorgplanning.

[12] Netwerken Palliatieve Zorg Fryslan. Stappenplan Advanced Care Planning (ACP) - Proactieve Zorgplanning. https://palliaweb.nl/netwerk-friesland/voor-zorgverleners/stappenplan-acp.

[13] Van der Plas A, Onwuteaka-Philipsen B, Willems D, Eliel M, de Wit ‐ Rijnierse M, Klinkenberg M (2019). Vroegtijdig spreken over behandelwensen (proactieve zorgplanning) in de eerste lijn.

[14] Glaudemans JJ, van Charante EM, Wind J, Oosterink JJ, Willems DL (2018). Experiences with approaches to advance care planning with older people: a qualitative study among Dutch general practitioners. BMJ Open.

[15] Nederlands Huisartsen Genootschap. NHG-Standpunt Toekomstvisie Huisartsenzorg NHG; 2009.

[16] Murray SA, Kendall M, Mitchell G, Moine S, Amblàs-Novellas J, Boyd K (2017). Palliative care from diagnosis to death. BMJ.

[17] Murray SA, Kendall M, Boyd K, Sheikh A (2005). Illness trajectories and palliative care. BMJ.

[18] Lattie EG, Asvat Y, Shivpuri S, Gerhart J, O’Mahony S, Duberstein P, Hoerger M (2016). Associations between personality and end-of-life care preferences among men with Prostate cancer: a clustering approach. J Pain Symptom Manag.

[19] Kittle K, Gaines B, Boerner K (2017). The role of personality in advance care planning. Innov Aging.

[20] Cain CL, Surbone A, Elk R, Kagawa-Singer M (2018). Culture and palliative care: preferences, communication, meaning, and mutual decision making. J Pain Symptom Manag.

[21] Epstein RM (2000). The science of patient-centered care. J Fam Pract.

[22] Curvers N, Pavlova M, Hajema K, Groot W, Angeli F (2018). Social participation among older adults (55+): results of a survey in the region of South Limburg in the Netherlands. Health Soc Care Community.

[23] Donkers E, Thewessen E (2010). Vijf visies op sterven op basis Van De Manier waarop patiënten over hun sterfbed denken, zijn ze in een paar categorieën in Te Delen. Dat is nuttige kennis voor wie deze patiënten in De Laatste fase van Hun Leven begeleidt. Medisch Contact.

[24] Berkel Fv, Metaal S. Sterven op je eigen manier: diversiteit in wensen & behoeften. Motivaction Intenational BV In: Transmuraal Netwerk Midden-Holland Project nummer K. 2008;1717.

[25] Schrijver T (2011). Kijk op sterven. Huisarts en wetenschap.

[26] Stuurgroup Passende zorg in. de laatste levensfase. Niles wat kan, hoeft. Utrecht; 2015.

[27] Fried TR, Bullock K, Iannone L, O’leary JR (2009). Understanding advance care planning as a process of health behavior change. J Am Geriatr Soc.

