# Supplementary material for: Adjusting advance care planning to older people’s needs: results from focus groups and interviews
Source: BMC Health Serv Res. 2024 Jan 10;24:51. doi: 10.1186/s12913-023-10491-x (PMC10782636; doi:10.1186/s12913-023-10491-x)
Supplement: Supplementary file 2 — Supplementary Material 2 [file 12913_2023_10491_MOESM2_ESM.pdf]

Supplement: Interview questions for focus groups and individual interviews

Preparation of focus groups and interviews

- Send out invitations including information about the study and the voluntary participation.
- Provide information letter. Participants sign the informed consent form for participation in the study and fill in the STEM questionnaire.

Interview questions (focus group session of about 2,5 hrs or individual interview)

Ask if the study may be audio-recorded. (The recording are deleted after the termination of the study). Read out a short description of an older person based on the STEM profile and introduce the following case:
A person comes out of hospital. He/she had covid-19 and has lost a lot in terms of fitness, also has a bad heart and incipient cataracts.
Then discuss the questions below:

1. What role does the *social network* of this person play in making important care decisions?
   Sub-questions:
   a. With whom does he/she talk about health and care decisions?
   b. Who may possibly make care decisions for him/her?
   c. Whom can he/she ask for help? Who takes care of him/her, if anyone?
   d. To what extent does he/she accept help from the network?
2. What role do *health professionals* of play in making important care decisions?
   Sub-questions:
   a. Which professionals does he/she like to talk to? Only the GP or also others?
   b. Does he/she only talk about health or also about future care wishes?
   c. What are the needs of him/her for having a good conversation and for making decisions about future care wishes?
3. What *sources of information* does he/she use concerning important decision about care and/or health?
   a. Select five relevant sources of information on the provided list of possible information sources.
   b. Ask for a top 3
   c. How would you prefer to be addressed?
4. What does he/she consider *important aspects* concerning care wishes?
   a. Extending life 0—10—20—30—40—50—60—70—80—90—100
   b. Maintaining independence 0—10—20—30—40—50—60—70—80—90—100
   c. Reducing pain 0—10—20—30—40—50—60—70—80—90—100
   d. Reducing other symptoms 0—10—20—30—40—50—60—70—80—90—100
5. What should a healthcare professional *absolutely not do* when discussing care wishes? What is perceived as annoying?

Summarize the key elements collected during the session describing a prototypical older person (reflecting one of the 5 STEM profiles).

6. Can you identify with this description? Is there anything, you would like to add or change?
